# A health inequality impact assessment from reduction in overweight and obesity

**DOI:** 10.1186/s12889-020-09831-x

**Published:** 2020-11-30

**Authors:** Anne Mette Bender, Jan Sørensen, Finn Diderichsen, Henrik Brønnum-Hansen

**Affiliations:** 1 Department of Public Health, University of Copenhagen, Faculty of Health Sciences, Section of Social Medicine, CSS, Øster Farimagsgade 5, Postbox 2099, DK-1014 Copenhagen K, Denmark; 2 Danish Centre for Health Economics (DaCHE), University of Southern Denmark, Odense, Denmark; 3 Healthcare Outcome Research Centre (HORC), Royal College of Surgeons in Ireland, Dublin, Ireland

**Keywords:** Social inequality, Obesity, Health impact assessment, Life expectancy, IHD, Stroke, Diabetes, Multi-morbidity, Education, Overweight

## Abstract

**Background:**

In recent years, social differences in overweight and obesity (OWOB) have become more pronounced. Health impact assessments provide population-level scenario evaluations of changes in disease prevalence and risk factors. The objective of this study was to simulate the health effects of reducing the prevalence of overweight and obesity in populations with short and medium education.

**Methods:**

The DYNAMO-HIA tool was used to conduct a health inequality impact assessment of the future reduced disease prevalence (ischemic heart disease (IHD), diabetes, stroke, and multi-morbidity) and changes in life expectancy for the 2040-population of Copenhagen, Denmark (*n* = 742,130). We simulated an equalized weight scenario where the prevalence of OWOB in the population with short and medium education was reduced to the levels of the population with long education.

**Results:**

A higher proportion of the population with short and medium education were OWOB relative to the population with long education. They also had a higher prevalence of cardiometabolic diseases. In the equalized weight scenario, the prevalence of diabetes in the population with short education was reduced by 8–10% for men and 12–13% for women. Life expectancy increased by one year among women with short education. Only small changes in prevalence and life expectancy related to stroke and IHD were observed.

**Conclusion:**

Reducing the prevalence of OWOB in populations with short and medium education will reduce the future prevalence of cardiometabolic diseases, increase life expectancy, and reduce the social inequality in health. These simulations serve as reference points for public health debates.

**Supplementary Information:**

The online version contains supplementary material available at 10.1186/s12889-020-09831-x.

## Background

In most countries including Denmark the educational difference in prevalence of obesity is growing [1], although developments have stabilized in some areas [2]. No country has yet been able to reverse the obesity epidemic [3]. In both high- and medium-income countries, strong correlation exists between socioeconomic status and obesity. Higher prevalence of obesity is found among those with shorter educational attainment [4]. The educational inequality in obesity has remained stable in recent years for the population of the Capital Region of Denmark including Copenhagen City [5]. The pathways that link education to overweight and obesity (OWOB) are not fully understood. However, research suggests that they are partly explained by limited availability of resources to encourage engagement in healthy lifestyles [6], as well as restricted access to health-promoting activities [7]. In addition, it is plausible that a part of the variation is related to differences in exposure to OWOB. Individuals growing up in low socioeconomic status households are less likely to complete higher education and more likely to be exposed to psychological distress and unhealthy lifestyles, compared to individuals from families with high socioeconomic status. Such variations have an impact on the risk of numerous diseases later on in adulthood [8].

Education can be used both as a measure of literacy and ability to attain new knowledge, including health messages, and is an important indicator of social position [6]. As obesity is strongly correlated with increased risk of numerous diseases, in particular cardiometabolic diseases [9], the prevalence of obesity-related diseases is much greater among individuals with shorter education.

Few quantitative studies have simulated the effects of population-based lifestyle interventions targeted at socioeconomically disadvantaged groups [10]. Health impact assessment (HIA) tools offer dynamic modeling of future population health [11–13] and can be used to model health effect changes in risk factors such as OWOB [14].

The aim of this study was to simulate the potential impact on the prevalence of cardiometabolic diseases and life expectancy (LE), for the population of Copenhagen with short or medium education with similar OWOB prevalence as those with high education. The scenario was modeled by equalizing (reducing) the prevalence of OWOB among those with short and medium education to the same level as the population with long education.

## Methods

### The DYNAMO-HIA model

DYNAMO-HIA is a Markov-type model, developed to simulate the health of a dynamically changing population. The model projects health effects related to changes in exposure to risk factors. It follows causal pathways modeled by relative risk between exposure to risk factors, incidence of diseases, and death. A risk factor may affect the risk of contracting several diseases, which are assumed to be causally correlated and linked by relative risks. During simulation, the sex- and age-specific transition probabilities between risk factor states determine the risk factor status of the simulated population and is updated in annual increments [11]. Counterfactual scenarios are used as benchmarks, in which the risk-factor prevalence is set at different levels (e.g. goals set by politicians or analysts). The results of these benchmark scenarios are compared to the reference scenario, and typically model the future risk factor prevalence as reflected by the historical development. The reference and counterfactual scenarios are modeled independently. Risk factor levels are defined at the beginning of the simulation period, while the health effects continue to develop over time. For each scenario, the output of the DYNAMO-HIA model includes sex- and age-specific simulated results, total number of incident and prevalent cases and disease clusters, total LE, and LE with and without diseases. The simulated effects of counterfactual scenarios are calculated as the absolute and relative difference to the reference scenario.

DYNAMO-HIA requires a wide range of input data, including:

- Population sex and age distribution (from population registers)
- Population age and sex specific disease incidence, prevalence and excess mortality from diseases (from population health registers)
- Projected number of newborns (from birth projections)
- Sex and age distribution of identified risk factors (from surveys, health examinations or registers)
- Relative risks assumptions linking risk factors to morbidity/mortality and disease to other diseases/mortality (from literature).

More detailed information on the DYNAMO-HIA methodology can be found in appendix S1 and [11].

### Definition of reference and counterfactual scenarios

In the last decade, studies have shown that the proportion of individuals with OWOB in Copenhagen has remained stable [5, 15]. We defined the reference scenario where the weight distribution by education group (short, medium, long) remained constant during the simulation period (2017–2040). The reference scenario is thus referred to as the *steady weight* scenario. A counterfactual scenario was created where the prevalence of OWOB in the population with short and medium education was similar to the population with long education. This *equalized weight* scenario assumed no difference in prevalence of OWOB by education groups. We stratified the population into three educational groups and made separate projections of the two scenarios for the populations with short, medium and long education. The results present the 2040 projections of LE at age 30 (total, without and with diseases), and prevalence of cardiometabolic diseases (IHD, stroke, diabetes and combinations of these).

### Input data sources

As our study population we retrieved register data for the population of Copenhagen for the period from 1st January 2000 to 31st December 2014 (2000, men = 241,715; women = 253,984). Anonymized data on education, diseases and death were available from Statistics Denmark for each individual linked through the national central person registration number. These data were analyzed with permission from the Danish Data Protection Agency.

Since most people have completed their education in their late 20s, our analysis considered individuals aged 30 years and older. Morbidity and mortality data for the three education groups were stratified in sex and one-year age categories. To avoid fluctuations related to small numbers, the Microsoft EXCEL Linest software was used to smooth the sex and age related data before they were imported into the DYNAMO-HIA model [16]. Beyond the general aging of the population and arrival of newborns, the DYNAMO-HIA model includes no population change due to migration. However, Copenhagen has a high level of (mostly national internal) immigration of people aged 20–30 years and emigration of people aged 30–45 years. This reflects people moving to Copenhagen for education and leaving once education is complete. To take account of such changes, the Statistics Denmark projections of the 2040-sex and age distributions (men = 365,480; women = 376,650) were multiplied by the smoothed sex and age-specific disease prevalence.

### Education

Individuals’ record of highest educational attainment based on the International Standard Classification of Education (ISCED 2011) was categorized into short education (primary and lower secondary education; < 2 years of vocational training/education; ISCED: 0–2), medium education (upper secondary and post-secondary education; 2–4 years of vocational education; ISCED: 3–4) and long education (tertiary education; > 4 years of education; academic degree; ISCED: 5–8) [17]. Since the future distribution of education is uncertain, we applied the 2017 educational distribution across the 30–40 year group.

### All-cause mortality

Dates of death during 2000–2014 were obtained from the Central Person Register.

### IHD, stroke and diabetes – incidence, prevalence and mortality

Data on IHD (ICD-10: I20-I25) and stroke (ICD-10: I60-I69, G45) were obtained from the Danish National Patient Register [18] and the Danish Register of Causes of Death [19]. Data on diabetes was obtained from the Danish Diabetes Register (DDR) [20]. The DDR data includes information on all persons registered with diabetes in any of the relevant national registers (e.g. hospitalization and prescription registers) [20]. Disease prevalence was defined as the number of individuals (per 100,000 inhabitants) with at least one record during the period 2000–2014. Incident rates were calculated as cases per person-year at risk, and included individuals with their first record of a diabetes diagnosis during 2000 to 2014 among individuals with no diagnosis of diabetes in the preceding 10 years.

### Body mass index

Self-reported 2013-questionnaire data on height and weight from the Copenhagen County Health Profile (*n* = 9265) [18] were used to calculate body mass index (BMI) (weight (kg)/height (m)^2^). Individuals were categorized as normal weight (BMI ≤24.9), overweight (BMI = 25–29.9) or obese (BMI ≥ 30). These data were available for 72.3% of the respondents. However, the response rate was 52.3% and required weighting for non-responses. Statistics Denmark provided weighted data according to sex, age, education, ethnicity and health for Copenhagen. More details on the weighting method can be found in the paper by Christensen et al. [21].

### Relative risks linking risk factors, diseases and mortality

We used sex and age specific relative risks from meta-analyses of international peer-reviewed scientific papers [22] and additional material from governmental reports [23]. We obtained relative risks (RR) linking OWOB to the included diseases, OWOB to all-cause mortality, specific diseases to death, diabetes to IHD [24], and diabetes to stroke [22] (see S1 for a list of RR).

## Results

**Fig. 1:**
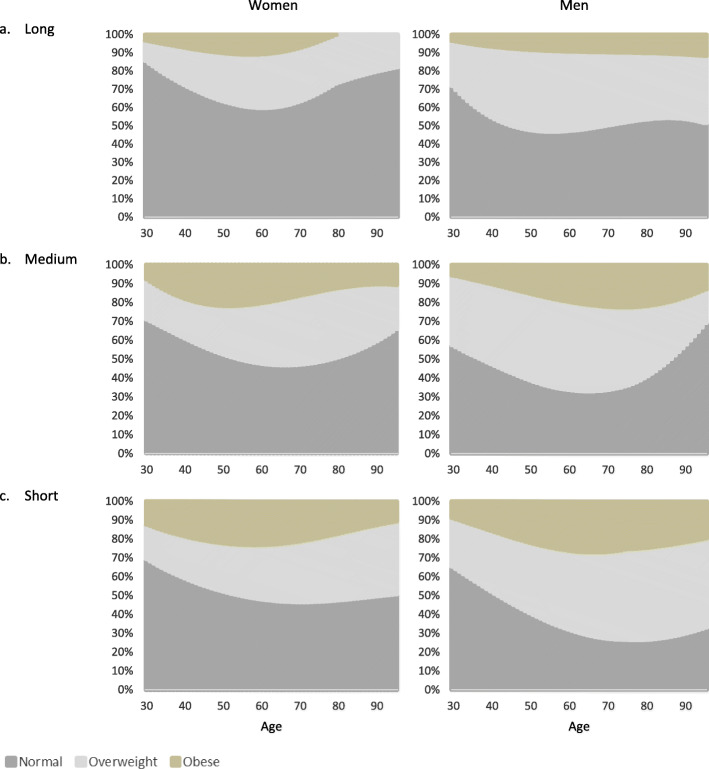
**a**-**c.** Steady weight class distribution across age and educational groups, men and women

Figures 1a-c. show that OWOB is more prevalent among men than women, and obesity is more prevalent among those with short and medium education compared to those with long education.

In the steady weight scenario, there is substantial difference in LE between education groups (Table 1). Men with long education have a longer LE by 6.4 years compared to men with short education (Table 1). Among women, this difference is 4.9 years. In the equalized weight scenario, the difference in LE between the education groups is reduced to approximately 3 months. A large reduction is observed in LE with diabetes (e.g. 1 year among women with short education) relative to small differences observed in LE with IHD and stroke.

**Table 1:** Simulated life expectancy (LE) in years at age 30 for three educational groups in 2040. Equalized weight scenario, difference in years (relative difference in %) compared to steady weight scenario

	Steady Weight Scenario, years			Equalized Weigh Scenario difference compared to Steady Weight Scenario, years (%)				
	Short education	Medium education	Long education	Short education		Medium education		Long education
**Men**								
Total LE	43.83	45.88	50.17	0.28	(0.7)	0.29	(0.6)	ref.
LE with IHD	6.13	6.37	6.41	−0.30	(−5.0)	−0.34	(−5.3)	
LE with diabetes	5.71	5.59	4.76	−0.61	(−10.7)	− 0.63	(−11.2)	
LE with stroke	4.26	4.66	4.86	−0.11	(−2.6)	−0.12	(−2.6)	
**Women**								
Total LE	48.61	50.17	53.50	0.27	(0.6)	0.20	(0.4)	ref.
LE with IHD	5.08	4.68	4.76	−0.36	(−7.2)	−0.29	(−6.3)	
LE with diabetes	5.63	5.15	3.73	−0.99	(−17.6)	− 0.83	(−16.1)	
LE with stroke	4.92	5.11	5.14	−0.20	(−4.1)	−0.19	(−3.7)	

Men have higher prevalence of cardiometabolic diseases than women (Table 2). In the equalized weight scenario, the prevalence of diabetes among women with short and medium education is reduced by around 12–13% and slightly less among men (8–10%). The reductions are also substantial for the prevalence of combinations with IHD & diabetes, diabetes & stroke. The prevalence is expected to decrease by 12% among women and 17% among men with short education. Similarly, reductions are expected in the prevalence of diabetes and multi-morbidity (combinations of diabetes, IHD and stroke) among men and women with medium education. The prevalence of IHD and stroke (and combinations thereof) is only slightly affected in the equalized weight scenario.

**Table 2:** Simulated disease prevalence per 100,000 inhabitants in the equalized weight scenario and difference compared to the steady weight scenario at age ≥ 30 years, across educational groups. Absolute cases (per 100,000 inhabitants) and relative difference (in %) at age ≥ 30 years, across educational groups

	Steady Weight Scenario, n			Equalized Weigh Scenario difference to Steady Weight Scenario, n (%)				
	Short education	Medium education	Long education	Short education		Medium education		Long education
**Men**								
IHD^1^	7145	6946	5954	−60	(−1)	−98	(−1)	ref.
Diabetes^1^	5463	4799	3591	− 484	(−8)	− 505	(−10)	
Stroke^1^	4439	4674	3763	98	(2)	164	(4)	
IHD + stroke	1105	1216	1040	−13	(−1)	41	(4)	
IHD + diabetes	1603	1490	1006	− 168	(−10)	− 137	(−8)	
Diabetes + stroke	2607	2324	1496	−416	(−14)	− 337	(− 13)	
Diabetes + stroke + IHD	1355	1294	809	− 271	(−17)	−200	(− 13)	
**Women**								
IHD^1^	5164	4533	1429	148	(3)	−1	(0)	ref.
Diabetes^1^	5379	4778	1114	− 800	(−13)	− 678	(− 12)	
Stroke^1^	5406	5376	1698	350	(7)	109	(2)	
IHD + stroke	963	866	309	109	(13)	−3	(0)	
IHD + diabetes	1485	1368	281	− 191	(−11)	− 221	(−14)	
Diabetes + stroke	1766	1371	313	−379	(−18)	− 295	(− 18)	
Diabetes + stroke + IHD	998	775	169	− 138	(−12)	−168	(− 18)	

^1^ Single diagnosis

The falling disease prevalence gradient by education is seen across all disease groups with the exception of stroke and the combination of IHD and stroke among men. The inequality in the prevalence of disease is considerably higher in women than men. The prevalence of diabetes and multiple cardiometabolic diseases among women with short education is approximately six times higher compared to women with long education. The most pronounced fall in inequality as an effect of the equalized weight scenario is seen for diabetes and combinations of two or three cardiometabolic diseases. With regard to stroke (and IHD among women) there is an increase in inequality of the disease prevalence.

## Discussion

### Main findings

This study shows that considerable health gains and reductions in health inequalities can be achieved if the prevalence of OWOB in populations with short and medium education is reduced. Preventing excess OWOB among individuals with short and medium education will increase both disease-free and total LE. Particularly, a reduction in inequality can be expected among individuals with diabetes and multi-morbidity. The inequality across obesity-related disease is particularly evident among women, while educational inequality in LE is similar for men and women.

In the scientific literature, gender differences and educational inequality in obesity-related health have not been addressed extensively. While analyses of gender differences and inequality in obesity are sparse and inconsistent [1, 25–28], most studies (with the exception of [29]) find higher relative inequality in cardiometabolic disease among women than men [30–33]. We also found these differences in this study. The larger difference in diabetes prevalence among women may be explained by the higher relative risk of diabetes from OWOB among women compared to men [23] (see S1, Supplementary File for a complete list of relative risks). Differences in body composition and fat deposition between men and women may also contribute to gender-dependent diabetes risk [34]. For example, there may be an overestimation of body fat mass based on BMI in men, who generally have more fat-free muscle compared to women [34].

Surprisingly, we see no substantial effect of the equalized weight scenario on IHD and stroke and this can be explained by two opposing mechanisms: a) longer LE among those with short education, which increases the risk of disease, and b) decreasing risk of these diseases due to lower OWOB. Therefore, higher levels of stroke and IHD should not be seen as a negative effect of the equalized weight scenario, but rather as an effect of longer LE.

### Strengths and limitations

One strength of the study is the use of data from national administrative registers on disease and mortality. These data have high validity and complete coverage of the population in Copenhagen. However, the information on disease includes only those diagnosed as part of hospital treatment as in- or outpatient, or by a general practitioner in the case of diabetes. Data on BMI were based on self-reported questionnaires, which were statistically weighted in order to approach representativeness for the reference population in terms of education. A newly published paper from the Danish Health Examination Survey (DANHES) showed a high correlation between self-reported height and weight and those from physical measurements [35], while a previously published study from Scotland found no differences in reporting of weight and height according to education status [36]. Reporting bias may vary between countries and over time, but we consider the potential effects of misclassification to be small. The validity of BMI scores as measures of OWOB has been discussed for decades [37]. Generally, there is a consensus that, in clinical research, BMI should be used in combination with other metrics of obesity, as BMI has limited sensitivity as a tool for diagnosing excess body fat. For instance, BMI can overestimate body fat among individuals with high muscle mass and underestimate body fat among those who have lost bone and muscle mass, as is the case among many elderly people [37]. However, in a public health setting, BMI classification provides an easy assessment of the distribution of OWOB in the population [38].

The DYNAMO-HIA model has many advantages, primarily related to the model’s ability to use information on disease levels and combine this with risk factor exposure, relative risks, and population demographics. As such, the model is assumed to make dynamic, real-life projections in contrast to regression-based models and quantitative risk assessment models, which are often static [16].

Drawbacks of the model relate to the assumption of constant relative risks over time, as well as uncertainties regarding the development in disease incidence from competing risk factors (and all other input data), which are general to all forecasting models. For instance, advantages in medication and medico-technologies are likely to increase the probability of survival, and therefore it is plausible that the disease-mortality relative risks will continue to decrease in the future [39].

Two papers have estimated the uncertainty of the model by running DYNAMO-HIA between five [40] and 100 [41] times, with different random seeds providing the 95% confidence intervals. The DYNAMO-HIA model does not provide estimated uncertainty as standard output, and while the above-mentioned analyses were carried out as comprehensive validation exercises, they did not consider uncertainty for all parameters. In general, the DYNAMO-HIA performed well in the estimation, but the uncertainty relied largely on the complexity of the epidemiological pathway, resulting in higher uncertainty regarding comorbidity and age groups/populations with high relative risk levels. In the future, developing tools that produce confidence limits related to dynamic simulation estimates would provide insight into the uncertainty of the results.

## Conclusions

This study is the first to use health impact assessment tools that combine advanced mathematical modeling and population data to assess the impact on health inequality from reduced education variation in OWOB. Diabetes is the main source of health inequality related to educational differences in OWOB. Preventing OWOB among people with short and medium education will reduce the future prevalence of cardiometabolic diseases, increase life expectancy and also reduce the social inequality in health. This underlines the relevance of specific policy implications regarding diabetes management targeted to populations with short education. These simulations serve as reference points for public health debates.

## Supplementary Information

**Additional file 1.**

## Data Availability

The data that support the findings of this study are available from the steering group of the Public Health Database. However, restrictions apply to the availability of these data, which was used under license for the current study, and so is not publicly available.

## Abbreviations

IHD: Ischemic heart disease
OWOB: Overweight/obesity
LE: Life expectancy
BMI: Body mass index
ISCED: International standard classification of education
HIA: Health impact assessment
RR: Relative risk
