# Supplementary material for: A health inequality impact assessment from reduction in overweight and obesity
Source: BMC Public Health. 2020 Nov 30;20:1823. doi: 10.1186/s12889-020-09831-x (PMC7706236; doi:10.1186/s12889-020-09831-x)
Supplement: Supplementary file 1 — Additional file 1. [file 12889_2020_9831_MOESM1_ESM.docx]

S1. **Relative risks included in the DYNAMO HIA model**

Due to the limited evidence available for children, a relative risk of one (1.0) used for individuals under the age of 20 years.

| All-cause mortality (1) | | | | | | | |
| --- | --- | --- | --- | --- | --- | --- | --- |
| **Age** |  | **0-20** | **21-49** | **50-59** | **60-69** | **70+** |  |
| Men | Normal weight | 1 | 1 | 1 | 1 | 1 |  |
|  | Overweight | 1 | 1.20 | 1.20 | 1.19 | 1.18 |  |
|  | Obese | 1 | 1.55 | 1.54 | 1.52 | 1.50 |  |
| Women | Normal weight | 1 | 1 | 1 | 1 | 1 |  |
|  | Overweight | 1 | 1.15 | 1.15 | 1.14 | 1.14 |  |
|  | Obese | 1 | 1.50 | 1.49 | 1.48 | 1.45 |  |
| BMI to Ischemic heart disease | | | | | | | |
| **Age** |  | **0-20** | **21-25** | **26-65** | **66-75** | **76-85** | **86+** |
| Men | Normal weight | 1 | 1 | 1 | 1 | 1 | 1 |
|  | Overweight | 1 | 1.25 | 1.35 | 1.30 | 1.25 | 1.20 |
|  | Obese | 1 | 1.70 | 2.00 | 1.80 | 1.60 | 1.40 |
| Women | Normal weight | 1 | 1 | 1 | 1 | 1 | 1 |
|  | Overweight | 1 | 1.25 | 1.35 | 1.30 | 1.25 | 1.20 |
|  | Obese | 1 | 1.70 | 2.00 | 1.80 | 1.60 | 1.40 |
| BMI to Diabetes | | | | | | | |
| **Age** |  | **0-20** | **21-25** | **26-60** | **61-75** | **76+** |  |
| Men | Normal weight | 1 | 1 | 1 | 1 | 1 |  |
|  | Overweight | 1 | 1.65 | 2.20 | 2.20 | 2.10 |  |
|  | Obese | 1 | 3.50 | 5.50 | 5.00 | 4.50 |  |
| Women | Normal weight | 1 | 1 | 1 | 1 | 1 |  |
|  | Overweight | 1 | 1.65 | 2.25 | 2.20 | 2.10 |  |
|  | Obese | 1 | 3.50 | 7.00 | 6.00 | 5.50 |  |
| BMI to Stroke | | | | | | | |
| **Age** |  | **0-20** | **21-25** | **26-64** | **65-69** | **70-79** | **80+** |
| Men | Normal weight | 1 | 1 | 1 | 1 | 1 | 1 |
|  | Overweight | 1 | 1.10 | 1.20 | 1.18 | 1.16 | 1.13 |
|  | Obese | 1 | 1.20 | 1.50 | 1.45 | 1.40 | 1.30 |
| Women | Normal weight | 1 | 1 | 1 | 1 | 1 | 1 |
|  | Overweight | 1 | 1.10 | 1.20 | 1.18 | 1.16 | 1.13 |
|  | Obese | 1 | 1.20 | 1.55 | 1.50 | 1.45 | 1.35 |
| Diabetes to IHD(2) | | | | | | | |
| **Age** |  | **0-20** | **21-55** | **56+** |  |  |  |
| Men | Diabetes | 1 | 2.66 | 1.93 |  |  |  |
|  | No diabetes | 1 | 1 | 1 |  |  |  |
| Women | Diabetes | 1 | 3.53 | 2.59 |  |  |  |
|  | No diabetes | 1 | 1 | 1 |  |  |  |
| Diabetes to stroke(3) | | | | | | | |
| **Age** |  | **0-20** | **21-55** | **56+** |  |  |  |
| Men | Diabetes | 1 | 2.00 | 1.80 |  |  |  |
|  | No diabetes | 1 | 1 | 1 |  |  |  |
| Women | Diabetes | 1 | 2.90 | 2.20 |  |  |  |
|  | No diabetes | 1 | 1 | 1 |  |  |  |

1. RR are based on scientific evidence from reviews and meta-analyses linking BMI to diseases and mortality. Documentation and data is available on www.dynamo-hia.org.

2. Yusuf S, Hawken S, Ounpuu S, et al. Effect of potentially modifiable risk factors associated with myocardial infarction in 52 countries (the INTERHEART study): case-control study. *Lancet Lond. Engl.* 2004;364(9438):937–952.

3. Barrett-Connor E, Khaw KT. Diabetes mellitus: an independent risk factor for stroke? *Am. J. Epidemiol.* 1988;128(1):116–123.
